# Safety and image quality of cardiovascular magnetic resonance imaging in patients with retained epicardial pacing wires after heart transplantation

**DOI:** 10.1186/s12968-021-00728-1

**Published:** 2021-03-15

**Authors:** Constantin Gatterer, Marie-Elisabeth Stelzmüller, Andreas Kammerlander, Andreas Zuckermann, Martin Krššák, Christian Loewe, Dietrich Beitzke

**Affiliations:** 1 Department of Biomedical Imaging and Image-guided Therapy, Division of Cardiovascular and Interventional Radiology, Medical University of Vienna, Währinger Gürtel 18-20, 1090 Vienna, Austria; 2 Department of Medicine II, Division of Cardiology, Medical University of Vienna, Vienna, Austria; 3 Department of Surgery, Division of Cardiac Surgery, Medical University of Vienna, Vienna, Austria; 4 Department of Medicine III, Division of Endocrinology and Metabolism, Medical University of Vienna, Vienna, Austria; 5 Department of Biomedical Imaging and Image-Guided Therapy, Medical University of Vienna, High-Field MR Centre, Vienna, Austria

**Keywords:** Cardiovascular magnetic resonance, MR safety, Cardiac transplantation, Image quality

## Abstract

**Background:**

Temporary epicardial pacing wires, implemented in patients during heart transplantation, are routinely removed before discharge. However, in some cases, these wires may remain in situ and are often considered as a contraindication for cardiovascular magnetic resonance (CMR) imaging in the future. Therefore, we aimed to provide data about safety and image quality of CMR in these patients.

**Methods:**

This is a report on a subpopulation out of 88 patients after heart transplantation that were included in a prospective cohort study and underwent multiple CMR in their post-transplant course. During CMR, patients were monitored by electrocardiogram and all examinations were observed by a physician to document potential adverse events. Additionally, image quality was assessed by an imaging specialist.

**Results:**

Nineteen of 88 patients included had temporary pacing wires in situ. These patients underwent a total of 51 CMR studies. No major adverse event and only one single, mild sensory event could be documented. All CMR studies showed preserved diagnostic image quality. Temporary pacing wires were visible in 100% of HASTE and cine sequences. In less than 50% of the examinations, temporary pacing wires were also visible in T1 and T2 mapping, short tau inversion recovery (STIR), and late gadolinium enhancement (LGE) sequences, without any impairment of image quality.

**Conclusions:**

With a low event rate of only one mild adverse event during 51 CMR examinations (2%), CMR appears to be safe in patients with retained temporary epicardial pacing wires after heart transplantation. Moreover, image quality was not impaired by the presence of pacing wires.

## Introduction

Patients after heart transplantation are at high risk for post-operative bradyarrhythmia, including atrioventricular block and sinus node dysfunction [1]. Thus, temporary pacing wires are routinely inserted in the right atrium (RA) and right ventricle (RV) after successful implantation of the donor’s heart. Furthermore, intermittent temporal pacing can facilitate the patients’ weaning process from cardiopulmonary bypass [2]. During the postoperative course, these wires are intended to be completely removed. However, in some cases, the wires have to be fixed at the myocardium (e.g. fixation by a suture to stop bleeding at the time of the insertion) or cannot be removed due to pericardial adhesion or overlap with sutures of the fascia. In these cases, pacing wires are usually cut below the skin level.

With these wires attached to the heart, patients are often excluded from magnetic resonance imaging (MRI) in the future [3]. As MRI utilization continuously increases and has a substantial impact on patient care, excluding these patients from MRI might potentially hinder a diagnostic work-up in the future [4, 5].

Causes for concern relate to potential neuromuscular voltage induction that could result in arrhythmias and movement, or heating of pacing wires with consequent tissue damage [6–8]. Preclinical data indicates that the greatest risk arises from the radiofrequency (RF) field due to the circularly polarized magnetic field associated with MRI devices. Higher amounts of energy may be deposited in the patient’s body by RF via abandoned pacemaker (PM) leads or pacing wires, resulting in heat burns and potentially inducing arrhythmic events [8, 9]. However, even using higher systemic absorption rates *ex vivo*, no myocardial damage could be observed so far [10]. Also there are several studies on patients undergoing MRI with retained temporary pacing wires published the that do not report any adverse events [3, 11–14]. However, the MR systems and scan regions in these reports were quite heterogeneous, and thus, did not allow a systematic comparison on this topic. To date, data about the safety of cardiovascular magnetic resonance (CMR) have been described only in a case report [12].

MRI and CMR with perrmanent pacemaker (PPM) systems including subcutaneous PPM generators is already a well-established procedure and as nowadays most of these systems are approved for CMR/MRI under specified conditions by the vendors [15]. In contrary to permanent systems temporary wires did not undergo systematic testing for being MR conditional as these “temporary” pacing wires are not intended to remain in the patient. Additionally, temporary pacing wires are not implanted in a standardized way, and therefore the shape and the length of the abandoned wires differ from patient to patient making it impossible to predict potential CMR interaction in the further course.

Another important aspect of CMR in contrast to other imaging regions is image quality when dealing with temporary pacing wires and conventional PPM remnants, as leads can significantly alter image quality due to artifacts [16]. Especially in CMR, these artifacts may conceal pathologies and could potentially influence diagnostic image quality, as well as quantitative values of mapping techniques.

Therefore, the aim of this study was to provide further evidence about the safety and image quality in a patient cohort with temporary pacing wires who were referred to multiparametric CMR in a prospective setting.

## Methods

Patients:

This subgroup analysis is part of an ongoing, prospective CMR cohort study of 100 patients after heart transplantation. It complies with the declaration of Helsinki and its amendments and was approved by the ethics committee of the Medical University of Vienna (IRB Nr.: 2012/2015). The inclusion criteria were: patients 18–99 years; and recent cardiac transplantation. Exclusion criteria were defined as the presence of abandoned implanted cardiodefibrilator (ICD) leads, claustrophobia, and pregnancy. Written, informed consent was obtained from each participating patient prior to study inclusion. The study was performed at a tertiary referral center that offered the entire spectrum of cardiovascular imaging and an organ transplant program, as part of EuroTransplant, which performs approximately 50 to 60 heart transplantations annually. Within this prospective cohort study, patients are scheduled for CMR 3, 12, 24, 36, 48 and 60 months after heart transplantation.

During heart transplantation surgery, temporary pacing wires (TME bifurcated, OSYPKA AG, Rheinfelden, Germany) were implanted at the RA and RV. The leads were then loosely folded to a loop and led intrapericardially to the xiphoid where they penetrated the skin to be connected to the extracorporeal PM. Epicardial wires that could not be removed were left in situ after being cut below the skin level. Patients who participated in this prospective cohort study and had retained temporary pacing wires were briefed about reporting any skin perceptions or heat sensations during the CMR examination.

Using routinely performed chest X-ray, computed tomography, and fluoroscopy, retained epicardial pacing wires were categorized according to their shape into “straight”, “C-shaped,” or “looped (antenna).”

All CMR examinations were performed on a 1.5T CMR scanner (Avanto Fit, Siemens Healthineers, Erlangen, Germany) with a maximum gradient strength of 45 mT/m and a maximum slew rate of 200 T/m/s. Specific absorption rate (SAR) limits for normal operation mode were 2w/kg and 4w/kg for first level mode, respectively. Two receive only coils (18-channel body array/32-channel integrated coil) were used. A standardized multiparametric CMR protocol was used to assess myocardial inflammation. The protocol included left ventricular structure and function, advanced tissue characterization by T1 and T2 mapping, conventional edema imaging, and scar assessment by late gadolinium enhancement (LGE) [17]. Details on the CMR protocol are listed in Table 1.

**Table 1:** Cardiovascular Magnetic Resonance (CMR) sequence pre-settings at 1.5T

Sequence	TI (ms)	TE/TR (ms)	Triggerpulse	Echo spacing (ms)	Flip angle (°)	Resolution (mm)	Layer-thickness (mm)	Readout bandwith (Hz/Pixel)
bSSFP Cine (SAx, 4Ch, 2Ch)		1.21/52.2		2.9	64	1.4 × 1.4	6	930
T_2_ HASTE		43/467	x2	3.32	160	1.6 × 1.6	5	781
T_1_-mapping (SAx,4ch) RR > 700ms)	180	1.13/279.84		2.7	35	1.4 × 1.4	8	1085
T_1_-mapping (SAx/4Ch) *RR < 700ms	180	1.01/263.55		2.4	35	1.9 × 1.9	8	1085
T_2_-mapping		27/193.27		2.5	70	1.9 × 1.9	8	1184
STIR	150	47/760	x2	6.74	180	1.5 × 1.5	8	235
Rest Perfusion (SAx)	115	1.07/193.72		2.2				
LGE (SAx,4Ch,3Ch, 2Ch)	(250) variable	1.19/700		2.9	50	1.5 × 1.5	8	781
Post-contrast T_1_-mapping (SAx,4Ch) *RR > 700	260	1.13/359.84		2.68	35	1.4 × 1.4	8	1085
Post-contrast T_1_-mapping (SAx,4Ch) RR < 700	260	1.01/241.12		2.43	35	1.9 × 1.9	8	1085

*Heart rate adapted; *bSSFP*, balanced steady state free precession; *SAx*, short axis;* 4Ch*, four chamber; *3Ch*, three chamber;* 2Ch*, two chamber; *RR*, RR-Interval (ECG); *STIR*, short tau inversion recovery; *LGE*, late gadolinium enhancement; *TI*, inversion time; *TE*, repetition time;* TR*, repetition time

All examinations were supervised by a physician and possible adverse reactions were documented. Patients were monitored using the CMR integrated electrocardiogram (ECG) gating device. Severe adverse events were defined as arrhythmias, self-reported heat sensations, hemodynamic instability, or any other possibly life-threatening complication. Mild adverse events were defined as every other event that did not meet the criteria for a severe adverse event and was clinically associated with a potential lead/CMR interaction. Furthermore, each CMR examination was assessed for image quality by two CMR specialists.

Statistics:

Descriptive statistics were performed for patient characteristics, image quality, and events. Continuous variables were reported as mean ± standard deviation if normally distributed, or as median ± interquartile range for non-normally distributed continuous variables. Categorical variables were described as counts and percentages. Cohen’s kappa was used to report interrater variability of image quality and visibility between two blinded readers.

## Results

### Patient characteristics

Overall, 88 patients were included in this ongoing prospective cohort study by July 2020. Nineteen of these 88 patients (21.6%) had retained temporary pacing wires in situ (42% female/ 58% male). Eleven patients had both the RA and the RV pacing wires in situ, eight patients only had the RA wire in situ. The reasons for heart transplantation in this sub-group were ischemic cardiomyopathy (n = 7), dilative cardiomyopathy (n = 9), Anderson-Fabry disease (n = 1), amyloidosis (n = 1), and valvular disease (n = 1). Most patients had repetitive CMR examinations (5 patient had four, 6 patients had three and 5 had two examinations respectively). One patient only had a single CMR examination at the time of analysis. Overall, a total of 51 CMR examinations were analyzed for safety and image quality. The median time from heart transplantation until the first CMR was 96 days (interquartile range: 88–119 days).

In one patient, the short tau inversion recovery (STIR) sequence was not added to the protocol. In another patient, LGE and post-contrast T1 mapping sequences were interrupted due to the onset of an allergic reaction to the contrast agent. All other scans were completed according to the study protocol.

### Visibility of temporary pacing wires and CMR image quality

Using chest X-ray, temporary pacing wires leads were visible in only 10 of 19 patients (53%). In eight patients, the leads were detected by chest or cardiac computed tomography (CT) scans, while, in one patient, the existence of retained wires was only self-reported, but could not be verified on any radiological images prior to CMR.

**Fig. 1:**
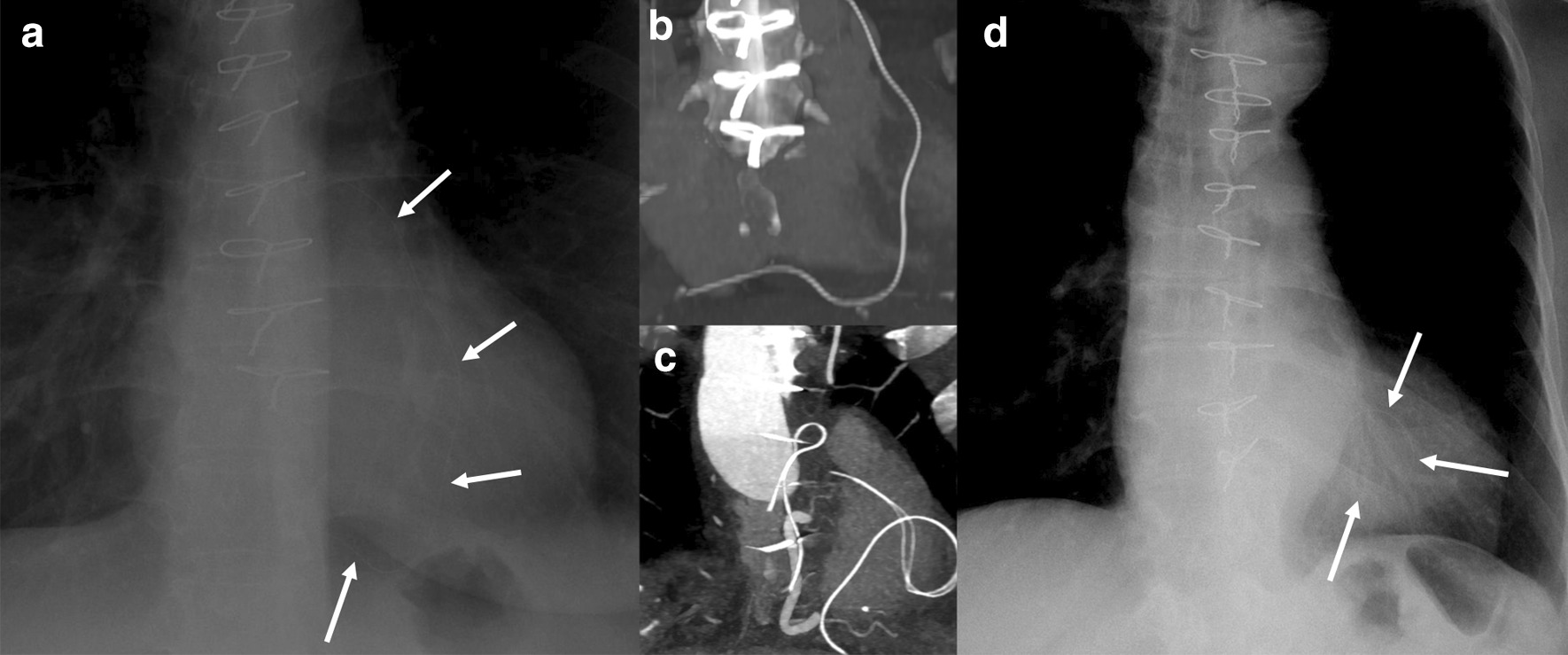
Representative chest x-ray (**a**, **d**) and maximum intensity projection images computed tomography (CT) (**b**, **c**) images of patients with retained temporary wires (C shaped configuration in **a**, **b**; looped configuration in **c**, **d**. Note the impaired visibility of the wires (white arrows) on the conventional chest X-Rays

The most frequent lead form, derived from other imaging modalities, was a loop (47%), followed by C-shaped (32%), and straight (21%). An overview of lead forms is displayed in Table 2. Figure 1 shows a representative cases of a C-shaped and looped retained pacing wire, hardly visible on chest X-Ray.

**Table 2:** Overview of patient characteristics, frequency and form of retained temporary pacemakers leads, and events

Characteristic	n = 19 (%)
Retained temporary PM leads, n (%)	19/88* (22)
Age, year	54 ± 15
Male patients, n (%)	11 (58)
CMR Studies	51 (91)
Form	
Loop	9 (47)
C-shaped	6 (32)
Straight	4 (21)
Adverse reactions	
Severe	0 (0)
Mild	1 (5)
Event rate	
CMR, n (%)	1/51 (2)

Numbers are given as mean ± standard deviation or count and (%)

*Total heart transplantation CMR study cohort; *PM*, pacemaker

**Fig. 2:**
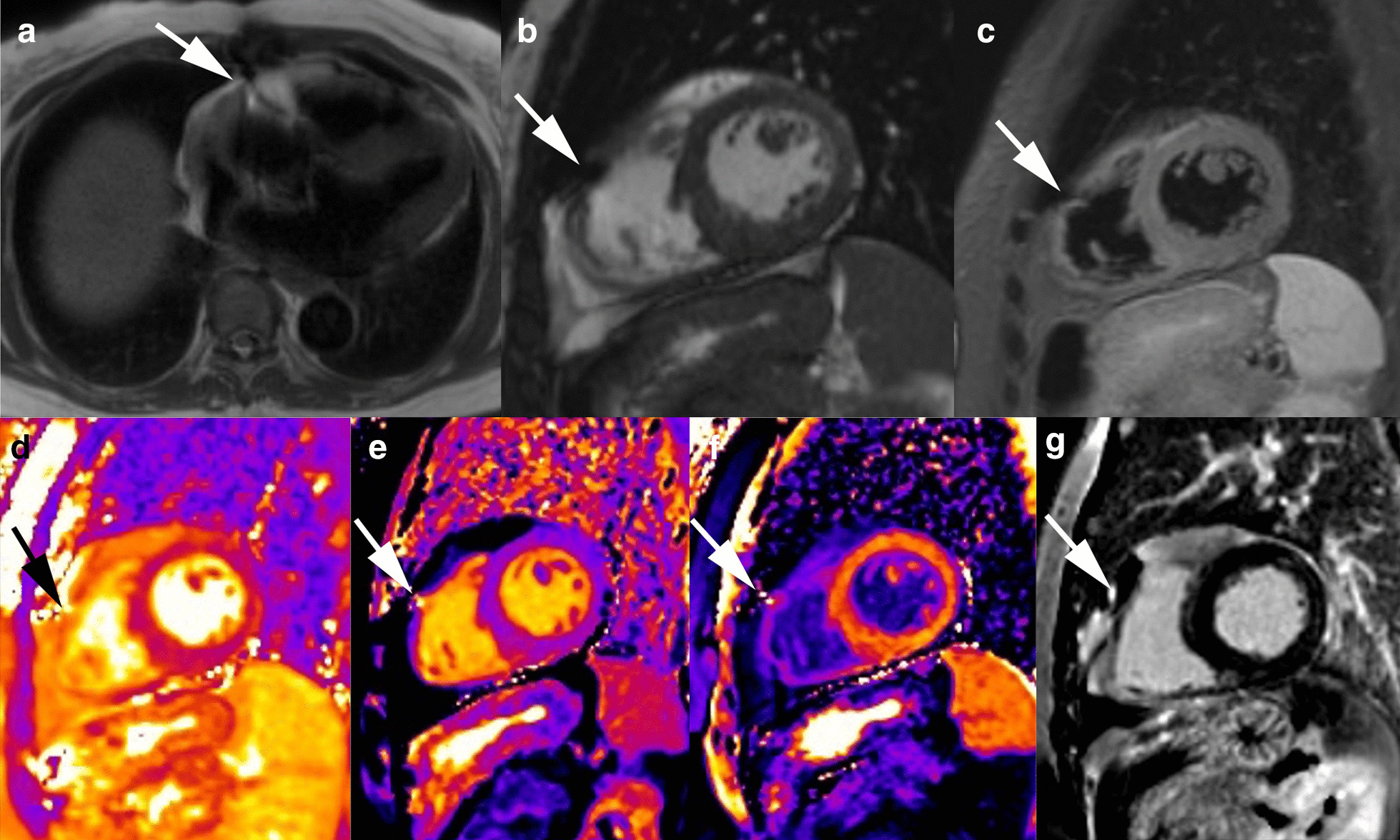
Preserved image quality of CMR in a patient with retained wires (white arrows). None of the sequences are obscured by artifacts (**a**–**g**)

Image quality was of diagnostic quality in all CMR studies. Although retained pacing wires were visible in all half Fourier single-shot turbo spin-echo (HASTE) sequences and 78% of cine sequences during CMR, diagnostic image quality was not degraded. Image quality in T1/T2 mapping sequences, and LGE images was also not impaired, as the wires could be detected in only 45% and 40%, respectively. A detailed overview of image quality and artifacts can be found in Table 3. Examples of image quality and artifacts are shown in Fig. 2.

**Table 3:** Visibility and image quality of temporary Pacemaker leads in CMR

Grading of image quality	n = 51(%)	n = 51(%)	n = 51(%)	n = 50(%)	n = 48(%)
	HASTE	Cine	T_1_ /T_2_ mapping	STIR	LGE
Not visible	0 (0)	11 (22)	23 (45)	18 (36)	14 (29)
Visible, no image quality impairment	51 (100)	40 (78)	23 (45)	23 (46)	19 (40)
Visible, impaired image quality	0 (0)	0 (0)	0 (0)	0 (0)	0 (0)
Not in field of view	0 (0)	0 (0)	5 (10)	9 (18)	15 (31)

Numbers are given as count and (%). *HASTE*, half-Fourier single shot turbo spin echo, *LGE*, late gadolinium enhancement; *STIR*, short tau inversion recovery

A good interrater agreement was found for all measures, ranging from 68% for mapping sequences to 82% for cine imaging (kappa from 0.46 to 0.57, p < 0.001 for all). Both readers agreed that leads did not impair image quality in any of the cases. An overview of agreement and kappa can be found in Table 4.

**Table 4:** Cohen’s Kappa for interrater variability of image quality

Measure	Agreement	Kappa	p
Cine	82%	0.48	< 0.001
Maps	68%	0.46	< 0.001
STIR	80%	0.57	< 0.001
LGE	71%	0.52	< 0.001

### Safety/adverse events

During 51 CMR examinations, no serious adverse event, such as arrhythmias, self-reported heating of the pacing wires, or severe pain, could be observed. One patient, with a C-shaped temporary pacing wire in situ, described a sensory event near the subcutaneous end of the retained lead during the second CMR, 12 months after heart transplantation. This event occurred during the HASTE sequence. The effect was reproducible, as it immediately disappeared once the sequence was discontinued, but returned as soon as it was restarted. No arrhythmic event or signs of skin irritation were observed. The CMR scan was stopped and the patient was transferred out of the CMR unit. The further course was uneventful; however, the patient was excluded from the study. All other CMR studies were completed uneventfully. With only one event of 51 CMR, the event rate was 2%.

## Discussion

The main finding of this study is that multiparametric CMR at 1.5T in patients with retained temporary pacing wires is associated with a low incidence rate of 2% mild adverse events. During a total number of 51 CMR scans in 19 patients, not a single serious adverse event was observed. Furthermore, the image quality of CMR is not altered by retained temporary pacing wires.

MRI and, in particular, CMR utilization is continuously rising, with an increase in use and an increasing number of clinical indications for all patient groups, not only in patients after cardiac transplantation [18, 19]. The use of MRI is reported to have an important impact on clinical decision-making, with added value for patient management [4].

Patients after cardiac surgery and, especially after cardiac transplantation, often present with metallic remnants, either after incomplete ICD lead extraction or with temporary pacing wires as in our cohort [20]. As a consequence, patients with retained wires are often prohibited from receiving MRI, thus possibly facing worse outcomes [3, 21]. Retained temporary pacing wires do not represent a regular medical implant, as the leads are intended to be removed after the operation. Therefore, these pacing wires are not tested for MR compatibility in a defined MR environment. In addition, the form of the wires within their epicardial course varies between patients, making it difficult to exactly predict MR-related complications. A further cause of concern may be the positioning of the patient with the wire in the center of the gradient and radiofrequency field as necessary in CMR. Thus the induction of heating and current might be more likely as if compared to other scan regions [22].

Overall, potential risks arise from interactions with multiple components of the MR system that should be taken into account separately prior to imaging in these patients. The static magnetic field (B0) may interfere with ferromagnetic implants by inducing translational force and torque. However according to the manufacturer the wires used in our cohort do not contain ferromagnetic components [23].

Relevant heating may be caused by the RF field resulting in thermal injury [23]. Kappus et al. showed that, in contrast to transvenous ICD and pacemaker leads, for which MR related heating up to 53.6°C was described *ex vivo*, no relevant heating of looped and/or straight temporary pacing wires could be found by *in vitro* testing using an infrared camera [9, 24]. Furthermore, an *ex vivo* CMR study of 12 swine hearts with implanted pacing wires provided no evidence of thermal damage to the myocardium, as evaluated in the histological examination after the CMR examination [10]. In this context it is also important that only a receive coil is used for standard CMR as the potential for heat induction over the wire is higher using a transmit/receive coil located close to the imaging center [23].

The time varying gradient magnetic field may be associated with (minor) heating of implants and neuromuscular excitation that may present as arrhythmic cardiac events [23]. Lead length and configuration, amplitude and phase of the electric field along the lead influence the current, which is potentially induced into the wires and eventually could lead to current induction, as well as additional heat elevation of the retained leads [8, 25, 26]. This theoretical risk of arrhythmia induction might even be higher if the retained wire is still connected to the RV and/or in case of a cardiac structural abnormality like a scar formation [23]. However, there are no reports available on arrhythmia induction by MR in the presence of retained pacing wires. Additionally, it may also be debatably whether a routine CMR protocol is able to induce voltages beyond the pacing thresholds for the atrial and ventricular leads respectively [27, 28]. These pacing thresholds are reported to increase over the time and therefore a (C)MR far after implantation may pose a lower risk for the patient [27].

Our findings are in line with previous reports that MRI and CMR with temporary pacing wires does not lead to serious lead related complications, thus supporting the hypothesis that CMR is safe in patients after heart surgery [3, 11–14, 29].

To date, only one case of an adverse reaction during CMR in this context has been reported in the literature. The patient, who experienced anginal symptoms during stress CMR, fully recovered and his symptoms were thought to be primarily caused by his underlying ischemic cardiomyopathy [13]. Therefore, and based on the data from this cohort, MRI, and particularly CMR, can safely be performed despite the presence of temporary right atrial and right ventricular pacing wires, with very low risk for these patients [30].

There are multiple case studies published, that describe non-MRI-related migration of the wires into various regions, including the right coronary artery, which caused an infarct, and into the ascending aorta [31, 32]. In addition, infectious complications, such as breast abscess formations, have also been reported [33].

Although various approaches have been pursued by different centers to detect retained wires before an MRI/CMR scan, including screening of previous radiological studies, or reviewing the patient’s history, in our cohort, multiple chest x-ray images in the anterior posterior view were obtained during the patients’ treatment at the intensive care unit and during routine follow up. However, in 53%, retained wires could be detected only on CT or fluoroscopy images.

When analyzing image quality, the impact of retained pacing wires on CMR image quality was negligible. No relevant artifacts could be observed, as the wires neither concealed any pathology nor hampered the use of mapping techniques or post-contrast images. In contrast to residual transvenous PPM and ICD leads, retained temporary pacing wires did not result in any relevant impairment of image quality [34, 35]. Interrater agreement was good, an excellent agreement was probably not reached as the artifacts caused by the pacing wires could often hardly be detected.

Limitations:

There are limitations of our study that hinder the transition of these results to general radiology. As all CMR examinations were performed on a 1.5T according to a standardized protocol, results cannot be easily extrapolated to other protocols that include sequences with a higher risk of wire-heating and voltage induction (e.g., diffusion-weighted imaging, prolonged turbo spin echo sequences), other scan areas with different coil settings, or other field strengths. Other limitations were the relatively small sample size and the fact that ECG rhythms were observed for arrhythmias during CMR, but could not be systematically analyzed subsequently. However, most of the patients in this analysis underwent multiple uneventful CMR.

## Conclusions

Repetitive CMR at 1.5T in patients with retained temporary right atrial and right ventricular pacing wires after heart transplantation or cardiac surgery appears to be safe and delivers preserved image quality. Therefore, CMR at 1.5T should be offered to these patients without restrictions.

## Data Availability

The datasets used and/or analysed during the current study are available from the corresponding author on reasonable request.

## Abbreviations

2Ch: Two chamber
3Ch: Three chamber
4Ch: Four chamber
CMR: Cardiovascular magnetic resonance
CT: Computed tomography
ECG: Electrocardiogram
HASTE: Half Fourier single-shot turbo spin-echo
ICD: Implanted cardiodefibrillator
LGE: Late gadolinium enhancement
MRI: Magnetic resonance imaging
PM: Pacemaker
PPM: Permanent pacemaker
RA: Right atrium/right atrial
RF: Radiofrequency
RV: Right ventricle/right ventricular
SAR: Specific absorption rate
SAx: Short axis
STIR: Short tau inversion recovery
